# Cold snap for cancer: cold-induced brown fat thermogenesis starves tumor growth

**DOI:** 10.1038/s41392-022-01284-5

**Published:** 2023-01-09

**Authors:** Ziqing Chen, Yibin Kang

**Affiliations:** 1 Department of Molecular Biology, Princeton University, Princeton, NJ 08544 USA; 2 Ludwig Institute for Cancer Research Princeton Branch, Princeton, NJ 08544 USA

**Keywords:** Cancer metabolism, Tumour immunology

In a recent study published in *Nature*, Seki et al. reported that cold-induced brown fat activation impedes glycolysis and reduces tumor growth in multiple cancers,^1^ deepening our understanding of the complex role of fat metabolism in cancer.

Cancer cells switch energy generation from effective Krebs-cycle and oxidative phosphorylation to inefficient aerobic glycolysis, a phenomenon known as the Warburg effect.^2^ Such adaptation dramatically increases glucose uptake rate, helping tumor cells overcome the energy shortage and providing a carbon source for anabolic processes. Approaches to cut off the glucose supply from the tumor microenvironment (TME), such as calorie-restricted diet, fasting, and metformin showed promising results in controlling tumor growth.^3^

In the *Nature* study, Seki and colleagues found that immunocompetent mice subcutaneously implanted with colorectal cancer cells experienced marked tumor suppression when mice were housed in 4 °C environment compared to those in 30 °C, with a striking 80% reduction in tumor growth on day 20 after tumor inoculation. Cold acclimatization led to improved survival of mice and significantly reduced tumor hypoxia, angiogenesis and tumor cell proliferation.

Such observation of cold-induced tumor suppression (CITS) was replicated in a wide range of transplanted tumors in mice, including fibrosarcoma, melanoma and pancreatic cancer, as well as two genetic models of spontaneous tumors (MMTV-PyMT breast cancer and *Apc*^*Min/+*^ intestinal adenoma). Both the *Apc*^*Min/+*^ intestinal tumor model and an experimental liver metastasis model of colorectal cancer (CRC) ruled out the possibility that CITS is an artifact caused by low-temperature contact of superficial subcutaneous tumors or mammary gland tumors.

Brown-adipose tissue (BAT), a specialized mitochondria-enriched “engine”, is the body’s first line of maintenance of thermoneutral ambient temperature in cold acclimatization through non-shivering thermogenesis (NST). The authors found that cold acclimatization causes morphological and molecular changes associated with BAT activation and white adipose tissue (WAT) browning. Furthermore, tumor implantation did not significantly affect cold-augmented BAT activation compared to non-tumor bearing mice. To investigate the glucose distribution during CITS, positron emission tomography-computed tomography (PET-CT) and ^18^F-fluorodeoxyglucose (^18^F-FDG) were applied to trace the glucose distribution during cold acclimatization. Interestingly, under thermoneutral conditions, ^18^F-FDG was mainly detected in the tumor and only modest signals were detected in BAT. In contrast, the ^18^F-FDG signals were barely detectable in the tumor, but markedly accumulated in the interscapular BAT (iBAT) under cold acclimatization.

**Fig. 1:**
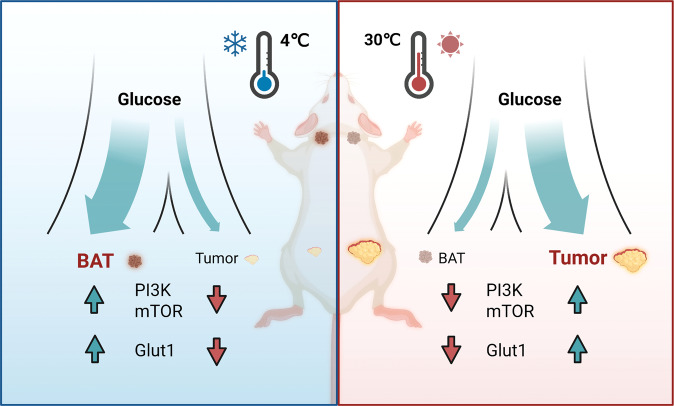
Cold-induced brown adipose tissue thermogenesis leads to tumor suppression by competing for supply of circulating glucose. In tumor-bearing mice under thermoneutral ambient temperature, more glucose is distributed to the tumor to support glycolysis and stimulate multiple metabolic pathways to facilitate tumor growth. Seki et.al report that brown adipose tissue activation during cold acclimatization favors glucose distribution to BAT and limits the tumor growth. Illustration is created with BioRender.com

To directly evaluate the functional role of BAT in CITS, the authors surgically removed the BAT in mice, and found a near complete loss of CITS effects, including tumor growth inhibition and suppression of tumor hypoxia and angiogenesis. Moreover, expression of several key glycolytic enzymes, glucose transporters (especially Glut1), as well as PI3K, AKT, and mTOR were inhibited in tumors undergoing cold exposure but were increased in the BAT of these mice, suggesting that glycolysis is suppressed in tumors but increased in BAT after cold exposure. These mice demonstrated decreased fasting blood glucose levels and improved insulin sensitivity. Additionally, the genetic deletion of UCP1, a key mitochondrial protein for thermogenesis in adipose tissue, mitigated the CITS effects. Intriguingly, the authors observed that CRC-tumor-bearing mice fed with high glucose experienced significantly increased PI3K pathway activation and GLUT1 expression, with abolished CITS effects even under cold environment. These data indicated that blood glucose competition between tumors and BAT is the key mechanism for CITS (Fig. 1). Underscoring the clinical relevance of their finding, the authors recruited 6 healthy young volunteers and confirmed BAT activation increased ^18^F-FDG uptake after 2–6 h of exposure to mild cold (16 °C) per day for 14 days. Most notably, BAT activation and significantly decreased glucose uptake in tumor was observed in a young Hodgkin’s lymphoma patient who was exposed to mildly cold ambient temperature (22 °C) for one week.

Fat metabolism has been noted to play an active role in supporting tumor growth and metastasis. Increased fatty acid oxidation (FAO) sustains ATP and NADH levels in tumor cells, and exogenous lipid sourcing is also critical for them to survive under hypoxic TME. Exogenous fat and lipid receptor CD36 has also been shown to promote metastasis.^4^ The current study revealed a tumor-suppressive role of cold-induced BAT activation in animal models. Whether cold exposure could prevent, arrest, or even reverse cancer progression remains to be validated in clinical trials in human patients. Specifically, carefully designed clinical trials are needed to evaluate which tumor type, stage, and treatment strategy may benefit from CITS. In addition, all the experiments were done with young mice which contain relatively more BAT compared with aged mice. It will be interesting to explore the CITS effect on aged mice, since cancer is more likely to happen in older people. In fact, a decline of BAT depots and activity is one of the hallmarks of aging, which may impair the CITS effect in older patients. Furthermore, this exciting study raised many new questions for future explorations. What is the role of immune cells in CITS? Whether this glucose redistribution could enhance or impair the immune system still remains unknown. Despite the decrease of CD45^+^ cells, which may contain both anti-tumor and pro-tumor immune cells, in the cold-exposed tumor, future works should be broadened to include single-cell multi-omic analysis of various organs and tissues to decipher the immune landscape of CITS and its potential impact on anti-tumor immunity. It is also important to understand the degree and duration of cold exposure needed to achieve CITS, and whether such treatment can be tolerated by cancer patients as they are already weakened by the disease and cancer treatments. As an alternative to cold treatment, targeting norepinephrine receptors in BAT using β3-adrenoceptor agonist such as CL-316 can induce thermogenesis effect similar to cold exposure. However, cardiovascular safety is of concern given the increase in heart rate and blood pressure observed with this class of medication. Finally, it remains to be seen tumor could develop resistance to cold treatment if they adopt an alternative source other than glucose.

Despite these remaining questions, the current study revealed a previously unknown anti-tumor aspect of brown adipose tissue, and suggested a potentially cost-effective and simple approach for cancer therapy.
